# Barriers to Successful Aging Among Older Women Living With HIV: A Qualitative Study

**DOI:** 10.1093/geroni/igaa057.1611

**Published:** 2020-12-16

**Authors:** Anna Rubtsova, Tonya Taylor, Gina Wingood, Igho Ofotokun, Deborah Gustafson, Marcia Holstad

**Affiliations:** 1 Emory University, Atlanta, Georgia, United States; 2 SUNY Downstate Health Sciences University, Brooklyn, New York, United States; 3 Columbia University Mailman School of Public Health, New York, New York, United States; 4 Emory University School of Medicine, Atlanta, Georgia, United States; 5 SUNY Downstate Medical Center, Brooklyn, New York, United States

## Abstract

Successful aging (SA) is the capacity of older people to thrive despite age-related changes and/or declines. Although our previous research found that older (age ≥50) women living with HIV (OWLH) can achieve SA, considerable barriers remain. The purpose of this qualitative study was to identify specific barriers to SA among OWLH. Our sample consisted of 29 OWLH recruited between October 2018 and March 2019 at two sites of Women’s Interagency HIV Study (WIHS): Atlanta, GA and Brooklyn, NY. These participants were assigned to either semi-structured interviews (N=17: 8 interviews in Brooklyn and 9 in Atlanta) or focus group discussions (FGD: 1 FGD in Atlanta with 5 participants, and 1 FGD in Brooklyn with 7 participants). Our FGD and Interview Guides included questions focused on barriers to SA. Participants were, on average, 58 years old (range 50-73), 86% Black, 83% single, and 62% with annual income ≤ $12,000. All interviews and FGD were transcribed and coded using MAXQDA software. We used thematic coding within constructivist approach. Several themes emerged identifying the following SA barriers: multiple chronic conditions and pain (e.g., arthritis, neuropathy); polypharmacy and side effects of HIV medications (“it’s wearing on me”); HIV-related stigma and loneliness (“I think my children would judge me if I would tell them I have it HIV”); substance use, giving up on yourself (“just sitting around, not doing anything”); and lack of access to resources and services (e.g., mental health providers, support groups). Our findings will help designing public health interventions promoting SA among OWLH.

